# Detection of SARS-CoV-2 in Hemodialysis Effluent of Patient with COVID-19 Pneumonia, Japan

**DOI:** 10.3201/eid2611.201956

**Published:** 2020-11

**Authors:** Ayako Okuhama, Masahiro Ishikane, Daisuke Katagiri, Kohei Kanda, Takato Nakamoto, Noriko Kinoshita, Naoto Nunose, Takashi Fukaya, Isao Kondo, Harutaka Katano, Tadaki Suzuki, Norio Ohmagari, Fumihiko Hinoshita

**Affiliations:** National Center for Global Health and Medicine, Tokyo, Japan (A. Okuhama, M. Ishikane, D. Katagiri, K. Kanda, T. Nakamoto, N. Kinoshita, N. Nunose, T. Fukaya, I. Kondo, N. Ohmagari, F. Hinoshita);; National Institute of Infectious Diseases, Tokyo (H. Katano, T. Suzuki)

**Keywords:** hemodialysis, respiratory infections, severe acute respiratory syndrome coronavirus 2, SARS-CoV-2, SARS, COVID-19, zoonoses, viruses, coronavirus, Japan, coronavirus disease

## Abstract

We report detection of severe acute respiratory syndrome coronavirus 2 RNA in hemodialysis effluent from a patient in Japan with coronavirus disease and prolonged inflammation. Healthcare workers should observe strict standard and contact precautions and use appropriate personal protective equipment when handling hemodialysis circuitry from patients with diagnosed coronavirus disease.

Since December 2019, coronavirus disease (COVID-19), caused by severe acute respiratory syndrome coronavirus 2 (SARS-CoV-2), has been a major health threat worldwide (*1*). Reports have been published on COVID-19 among patients receiving hemodialysis (*2*), but none have evaluated whether HD effluent is infectious. In addition, handling of hemodialysis circuitry is not mentioned in US Centers for Disease Control and Prevention (CDC) guidelines for COVID-19 infection control and prevention in dialysis facilities (*3*). We report detection of SARS-CoV-2 RNA in hemodialysis effluent from a patient with COVID-19 pneumonia and prolonged inflammation.

The patient, a 79-year-old man with end-stage renal disease (ESRD) due to IgA nephritis, had been receiving maintenance hemodialysis 3 times per week for 12 years. Six days before admission, he started having a fever and cough. Four days later, he had a nasal swab test for SARS-CoV-2 RNA. Quantitative reverse transcription PCR (qRT-PCR) (*4*) of the patient’s specimen was positive, and he was admitted to the hospital. At admission, his body temperature was 37.7°C and oxygen saturation was 98% on room air. Multiple bilateral patchy ground glass opacities (GGO) were observed on the patient’s chest computed tomography (CT) scan (Figure, panel A). Blood test results showed C-reactive protein (CRP) of 8.8 mg/dL and leukocyte count of 4,470 cells/μL. Although we started him on hydroxychloroquine (200 mg 2×/d) and azithromycin (500 mg 1×/d), he had a fever (>38.0°C) on day 2 of his hospitalization. A follow-up chest CT on hospitalization day 5 showed worsening COVID-19 pneumonia and expanding GGO areas (Figure, panel B). 

**Figure:**
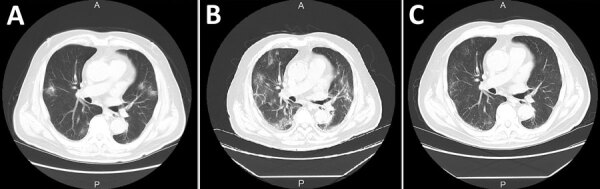
Chest computed tomography (CT) scan of a patient on hemodialysis diagnosed with positive reverse transcription PCR for severe acute respiratory syndrome coronavirus 2 in hemodialysis effluent, Japan. A) Chest CT at day 1 of hospitalization showing bilateral patchy ground glass opacities (GGO). B) Chest CT from day 5 of hospitalization showing worsening coronavirus disease 2019 (COVID-19) pneumonia with GGO expansion. C) Chest CT on hospitalization day 16 showing improvement of COVID-19 pneumonia; the patient was discharged on this day. A, anterior; P, posterior.

During the patient’s hospitalization, we administered hemodialysis by using a polysulfone membrane dialyzer in a private depressurized room with dedicated machines. We tested hemodialysis effluent for SARS-CoV-2 on day 2. PCR results showed SARS-CoV-2 RNA of 157.9 copies/μL with cycle threshold (C_t_) values of 38.3 at 1 hour after starting hemodialysis but were negative on effluent collected at 2 hours. Because the patient’s fever persisted and CRP levels remained high, on hospitalization days 9, 11, and 15 we performed direct hemoperfusion by using a β2 microglobulin adsorbent column (Lixelle-DHP) to absorb cytokine. On hospitalization day 10, the patient became afebrile and CRP began decreasing until it reached 5.9 mg/dL on hospitalization day 15. On hospitalization day 16, chest CT showed markedly improved pneumonia (Figure, panel C), and the patient was discharged (Table).

**Table:** Clinical course and quantitative reverse transcription PCR results for severe acute respiratory syndrome coronavirus 2 RNA in patient receiving hemodialysis, Japan*

Day after symptom onset	Hospitalization, d	Temperature, °C	Event			CRP, mg/dL	Dialysis	Specimens tested for SARS-CoV-2 by qRT-PCR‡			
				Medication†				Nasal swab	Blood	Effluent, time collected	
				AZM	Hydroxy					1 h	2 h
1		37.3									
2		37.2									
3		37.3									
4		37.3	Clinic					18.8 (NA‡)			
5		37.7									
6		39.0									
7	1	38.8	Chest CT	N	N	8.8	–				
8	2	38.4		Y	Y	9.0	Y	29.6 (1,080.6)§	ND§	38.3 (157.91)	ND
9	3	38.7		Y	Y	–	–	–			
10	4	38.7		Y	Y	14.0	–	–			
11	5	37.4	Chest CT	N	Y	15.0	Y	–			
12	6	37.0		N	Y	–	–	–			
13	7	37.2		N	Y	–	–	–			
14	8	37.0		N	Y	–	–	–			
15	9	36.9		N	Y	14.4	Lixelle-DHP	–			
16	10	37.0		N	N	–	–	34.3 (NA‡)			
17	11	36.9		N	N	–	Lixelle-DHP	ND			
18	12	36.9		N	N	13.7	–	ND			
19	13	36.8		N	N	–	–	–			
20	14	36.6		N	N	–	–	–			
21	15	36.7		N	N	5.9	Lixelle-DHP	–			
22	16	36.7	Chest CT, discharge	N	N	–	–	–			

*AZM, azithromycin; CRP, C-reactive protein; CT, computed tomography; Hydroxy, hydroxychloroquine; Lixelle-DHP, direct hemoperfusion using a β2 microglobulin adsorbent column; NA, not available; ND, not detected; qRT-PCR, quantitative reverse transcription-PCR; –, not done. †We prescribed azithromycin, 500 mg 2 times/d from day 1 to 3 because it was 1 of the potentially effective treatment regimens at the time. We also prescribed hydroxychloroquine 200 mg 2 times/d and initially planned to use it for 10 d in total, but the patient’s liver function tests (LFTs) became elevated during the course. We suspected side effects of hydroxychloroquine and stopped it on day 9. His LFTs returned to normal afterwards. ‡Results for SARS-CoV-2 shown as cycle threshold values (Viral load, copies/μL). Viral loads were not available because PCR was performed at an outside commercial laboratory where they did not report these results. The same PCR method was used (*4*) at both National Institute of Infectious Diseases (NIID), Japan, and the outside laboratory. HD effluent was collected at 1 hr and 2 hr into hemodialysis. §PCR test was performed at NIID, Japan where they report viral loads.

Our case highlights 3 things. First, inflammation and clinical symptoms of COVID-19 can persist in patients on hemodialysis. COVID-19 is thought to progress in a 2-stage manner: viral replication and hyperinflammation (*1*). Hyperinflammation starts 7–10 days after symptom onset and involves extensive lung areas. This patient’s fever persisted for >13 days, with pneumonia and CRP worse at 11 days after fever onset. Hyperinflammation appeared to progress slower and be maintained longer than in patients who are not receiving hemodialysis, which might be related to immune system dysfunction in patients with ESRD (*5*). Second, although SARS-CoV-2 RNA has been detected in various clinical specimens (*6*,*7*), our case demonstrates it also can be detected in hemodialysis effluent, even though we did not detect SARS-CoV-2 RNA in blood, as noted in a previous case (*6*). We hypothesized that only a small amount of fragmented RNA might pass through the dialysis membrane at the start of hemodialysis, but no marked fragments remain in the blood as a session progresses. Third, our case suggests Lixelle-DHP can have therapeutic effects for patients on hemodialysis. Although we did not measure the patient’s predialysis and postdialysis cytokine levels, use of a blood purification technique might alleviate the effects of cytokine in COVID-19 pathophysiology due to its proven effect in reducing plasma cytokine levels in general (*8*).

Our report has several limitations. First, we did not confirm the duplicability of PCR results of hemodialysis effluent. We performed PCR only once and did not reevaluate the same specimen, even though the C_t_ was high. Second, the infectiousness of hemodialysis effluent is unclear. Its viability should be quantified by endpoint titration on authorized cell lines, as previously reported (*9*). Third, this is a single case report. Despite these limitations, we cannot underestimate the infectiousness of hemodialysis effluent. We performed dialysis in a private room with dedicated machines. We also conducted strict standard and contact precautions when handling HD circuitry, following CDC recommendations for preventing transmission of hepatitis B virus infection among patients on HD (*10*). 

In conclusion, we report positive qRT-PCR results for SARS-CoV-2 RNA from hemodialysis effluent in a patient receiving renal dialysis. The clinical course of our patient was characteristic of the persistent inflammation of COVID-19 and shows the potential effectiveness of Lixelle-DHP as a treatment in patients on hemodialysis. Our case indicates that strict standard and contact precautions are essential when handling hemodialysis circuitry of patients with COVID-19. As more patients on hemodialysis contract SARS-CoV-2, we expect further studies on infection control and prevention in dialysis facilities and on the effectiveness of Lixelle-DHP in treating patients with COVID-19.
